# Combined Exercise and Education Program: Effect of Smaller Group Size and Longer Duration on Physical Function and Social Engagement among Community-Dwelling Older Adults

**DOI:** 10.14283/jarlife.2023.10

**Published:** 2023-07-25

**Authors:** S. Yamamoto, D. Ishii, K. Ishibashi, Y. Okamoto, K. Kawamura, Y. Takasaki, M. Tagami, K. Tanamachi, Y. Kohno

**Affiliations:** 1 Ibaraki Prefectural University of Health Sciences, Ibaraki, Japan; 2 Department of Cognitive Behavioral Physiology, Chiba University Graduate School of Medicine, Chiba, Japan; 3 University of Tsukuba Hospital, Tsukuba, Japan; 4 Osaka University, Osaka, Japan; 5 Keio University, Tokyo, Japan; 6 Tokyo Metropolitan University, Tokyo, Japan

**Keywords:** Community-dwelling older adults, exercise, educational program, physical function, social engagement

## Abstract

**Background:**

Exercise, education, and social engagement are critical interventions for older adults for a healthy life expectancy and to improve their physical function.

**Objective:**

To conduct a combined exercise and education (CEE) program for improved social engagement and physical function of older adults.

**Design:**

Based on a short-term program we conducted in our previous study, in this study, the program was conducted for half the number of participants of the earlier study but for a longer duration.

**Setting:**

A community of older adults in Ami, Japan, was the setting of the study.

**Participants:**

23 healthy older adults >65 years living in the community were the participants in the study.

**Interventions:**

Five 80-minute sessions conducted once in two weeks comprised 60-min exercise instruction and 20-min educational lectures per session on health. We examined the improvement in physical and social engagement before and after participation. Physical function and health-related questionnaire data were collected before and after the program.

**Results:**

Data analysis from 15 participants showed improved physical performance but no effect on social engagement.

**Conclusions:**

A higher program frequency, rather than program duration, may be vital to improving exercise performance and social engagement and maximizing the effects of high group cohesion in small groups. Further studies are needed to develop more effective interventions to extend healthy life expectancy.

## Introduction

**J**apan is currently faced with the various challenges necessitating adaptation to one of the highest rates of aging worldwide (1), one among which is addressing the health problems associated with aging. To extend the healthy life expectancy of older adults, developing effective preventive methods is essential.

Exercise is a crucial intervention for improving physical function and life expectancy in older adults (2-8), apart from educational programs that increase patient adherence to treatment (9) and demonstrate how patient education improves medication adherence rates.

Social engagement is another critical factor in extending healthy life expectancy in older adults, with higher social engagement reported to maintain cognitive function, reduce depressive symptoms, and lower the risk of dementia in community-dwelling older adults (10-14).

In a previous study, we have shown that a combined exercise and education (CEE) program (one 80-min session /week for five weeks) can improve the physical function and social engagement of older adults in a community-dwelling (15). However, the improvement in social engagement resulting from the CEE program was not sustained over a long period (lasting only a month after the last session), suggesting that a short-term intervention program cannot maintain increased social engagement and may require further involvement, such as scheduling opportunities for participants to gather regularly. Further, group cohesiveness often is greater in smaller groups (16), suggesting that a smaller group program may maintain increased social engagement.

Therefore, in this study, we examined the effects of a CEE program conducted for a longer period with half the number of participants on the physical and social engagement of community-dwelling older adults.

## Materials and Methods

### Participants

Participants were recruited in the same manner as in the previous study (15) through advertisements placed in town newsletters (Ami, Japan; population approximately 49,000) and distributed to existing mailing lists of the community site at which the study lectures were to be held. The advertisement mentioned that exercise, health condition measurements, and educational lectures would be included in the program.

Twenty-three healthy older adults aged >65 years who responded to the advertisements were assessed for eligibility and enrolled. The inclusion criteria for this study were the same as those of our previous study (15): the participants had to be (a) able to walk without the use of a cane or any other assistive device, (b) independent in activities of daily living (ADL), and (c) able to communicate. Those with cognitive decline were excluded, and participation was purely voluntary. The participants were briefed on the study instructions and requested to provide written informed consent to participate. The study was performed in accordance with the Declaration of Helsinki and was approved by the Ethics Committee of the Ibaraki Prefectural University of Health Sciences (approval no. e195).

### Experimental design and procedure

The frequency and total duration of the sessions differed from that of our previous study (15), but the length of each session was the same. Participants enrolled in one 80-minute session every two weeks (one week between the first and second sessions) for seven weeks. In the first session, each participant was given baseline measures of physical function (physical performance, body weight, height of body) and asked to respond to a health-related questionnaire. The second to fourth sessions consisted of 60 minutes of exercise instruction and a 20-min lecture about a health-related topic (comprising one session of the CEE program, described below). At the fifth/last session, post-intervention measurements of the above-mentioned aspects of physical function were conducted, and the participants again filled out the questionnaire about health-related matters.

### The combined exercise and education program (CEE program)

In the CEE program, participants received 60 minutes of exercise instruction and 20 minutes of educational lectures per session. Exercise instruction consisted of warm-up stretching (10 minutes), strength training (20 minutes), and balance exercises (10 minutes) followed by dual exercise (combined mental/physical exertion, 20 minutes). The educational lecture was presented in intervals between exercises. In the educational aspect of the program, lectures about health-related topics (the importance of maintaining muscle strength, nutritional intake, fall prevention, community development, and the impact of social participation on well-being) were presented by a physical therapist under the supervision of specialists in nutrition and gerontology.

### Baseline and post-CEE program assessments

The baseline and post-CEE programs relating to body weight measurement, skeletal muscle mass index, body mass index (BMI), physical performance, and health-related questionnaire matters were carried out by experienced physical and occupational therapists.

Body weight and height were measured, and BMI was calculated. For skeletal muscle mass index, body composition measurements were carried out by the bioelectric impedance method with a body fat analyzer (Inbody Dial H20B, Inbody Japan, Tokyo). The skeletal muscle mass index was calculated as follows: skeletal muscle mass [kg] / square of height [m].

For physical performance measurements, standardized protocols were used for the measurement of the 30-sec chair stand test (CS-30), and the timed up-and-go (TUG) test (17, 18). To measure grip strength, the participants’ dominant hand was used to measure maximum grip strength using a Smedley-type grip strength dynamometer (T.K.K.5401, Takei Scientific Instruments, Japan). The higher of the two measurements was used for analysis.

In the questionnaire about health-related matters, each participant’s level of social engagement, mobility, and fear of falling was assessed with the Elderly-status Assessment Set (E-SAS) developed by the Japanese Physical Therapy Association (19, 20). The E-SAS is based on the Lubben Social Network Scale-6 (LSNS-6) (21), the Life-Space Assessment (22), and the Falls Efficacy Scale (23). The number of times they were made to exercise per week and the number of falls per year was also noted for each participant before and after the 7-week program. For more detailed information, see our previous study (15).

### Statistical analysis

Data distribution was assessed using the Shapiro–Wilk normality test. The Wilcoxon signed-rank test was performed to compare the median value of the TUG, fear of falling, and number of falls per year before and after the CEE program because data were non-normally distributed. When the data were normally distributed, paired t-test was performed. Statistical significance was set at p < 0.05. All analyses were conducted with SPSS® 27.0 for Windows (SPSS, Inc., Chicago, IL, USA). The sample size was calculated after conducting the study using data from the primary outcomes CS-30, grip strength, and LSNS-6, as this information is necessary for planning the next study. The input parameters were effect size (calculated with data) = 1.67, 0.96, 0.08; correlation (calculated with data) = 0.81, 0.96, 0.84; error probability = 0.05; and power = 0.8; G* Power 3.1.9.7 for Windows (24, 25) was used.

### Multiple imputation

There were missing values for 1.5% of the measured parameters. We used multiple imputations (MI) as a statistical plan to account for missing data values. MI is a procedure used to replace missing values with other plausible values by creating multiple filling-in patterns to avert bias caused by missing data. In the present study, we replaced each missing value with a set of substituted plausible values by creating 50 filled-in complete data sets using MI by the chained equation method (26). In the imputation process, the following covariates were used to create 50 complete datasets: age, CS-30, TUG, grip strength, skeletal muscle index, number of exercise sessions per week, number of falls per year, LSA, FES, and LSNS-6. Multiple imputation analyses were conducted with R version 3.5.1 (27).

## Results

We evaluated 23 participants (10 males and 13 females) in the first session. Of these, 17 participants (8 males and 9 females) participated in the fifth session (6 dropouts). Finally, data from 15 participants (6 males and 9 females) who participated in the first and fifth sessions were analyzed. Baseline characteristics of participants and changes in assessment parameters post-CEE program are shown in Table 1. The mean values of CS30 and grip strength after the CEE program were significantly higher than those before the CEE program (t(14) = 4.737, p < 0.001, d = 0.535, and t(14) = 2.671, p = 0.018, d = 0.114, respectively). However, the skeletal muscle mass index after the CEE program was significantly lower than that before the CEE program (t(14) =3.329, p = 0.005, d = 1.445).

**Table 1.** Baseline characteristics of participants and changes in assessment parameters

	Baseline	Post-CEEP	p
Males / females	6 / 9	-	-
Age, years	70.7 ± 8.8	-	-
Body weight, kg	56.1 ± 7.5	55.6 ± 7.6	0.085
Skeletal muscle index, kg/m^2^	8.9 ± 0.5	8.7 ± 0.6	0.005*
BMI, kg/m^2^	22.4 ± 2.1	22.3 ± 2.3	0.319
Physical performance			
CS-30 test, times	21.3 ± 4.0	26.73 ± 5.5	< 0.001*
TUG test, sec	5.6 (5.5–5.9)	5.3 (4.9–6.2)	0.378
Grip strength, kg	30.7 ± 5.4	32.4 ± 5.8	0.018*
Health-related questionnaire:			
LSA	92.4 ± 14.1	90.5 ± 14.5	0.566
LSNS-6	16.7 ± 3.7	16.9 ± 4.4	0.814
FES	38 (34–40)	40 (35–40)	0.103
Exercise, times/week	2.9 ± 1.7	3.1 ± 1.1	0.550
Fall, times/year	0 (0–0)	0 (0–1.0)	0.180

The data are (mean ± std. dev.) or median (interquartile range). CS-30 test: 30-sec chair stand test, FES: Falls Efficacy Scale, LSA: Life-Space Assessment, LSNS-6: Lubben Social Network Scale-6, TUG: Timed up-and-go test. *p < 0.05

No significant differences were found before and after the CEE program in body weight, BMI, TUG, LSA, FES, the number of times of exercise, number of falls per year, and social engagement (p > 0.05).

We calculated the sample size after conducting the study, using data from the primary outcomes CS-30, grip strength, and LSNS-6, as this information is necessary in planning the next study. The results revealed the need for 4, 6, and 927 participants in each group, respectively.

## Discussion

In this study, we investigated the effects of the CEE program with a longer period and half the number of participants than our previous CEE program on the physical and social engagement of community-dwelling older adults (15). The major findings of this study were as follows: (1) the current CEE program (with an extended period and half the number of participants than our previous CEE program) improved their physical performance, (2) but had no effect on their social engagement.

The previous CEE program (one 80-min session 1x/ week for 5 weeks) increased exercise habit (>3 times/ week) and improved the physical function of elderly individuals living in a community dwelling (15). In this study, we found that the current CEE program did not increase exercise habits, but it did improve physical performance. The participants in the present study exercised >3 times/week, including in our program. Our current and previous results suggest that physical performance may be improved by an exercise habit of at least three times per week and participation in the CEE program at least once every two weeks.

We have previously shown that the CEE program (80 minutes x 1 session/week x 5 weeks) improves social engagement of older adults living in a community dwelling (15). However, this improvement in social engagement with the CEE program was not found to sustain in the long term (one month after the last session), suggesting that a short-term intervention program may not be sufficient to sustain improvement in social engagement and that additional engagement, such as setting up opportunities for participants to meet regularly may be necessary for longer-term improvement in social engagement. Nevertheless, in the current study, the CEE program with a longer period than our previous CEE program did not change the social engagement of community-dwelling older adults, suggesting the need for a higher program frequency rather than a longer period of the program for improving social engagement.

Perceptions of task and group cohesiveness have been reported to be greater in smaller groups (16). Members of cohesive groups are more likely to readily join and remain in the group (28). A review of studies on cohesiveness in sports teams and exercise groups found that team success, collective efficacy, and group communication are positively related to performance (29). The present study consisted of a small number of participants to increase group cohesion, but the effect of this in improving exercise performance was lesser than in previous studies; social engagement also showed no increase. These previous studies and our current result suggest that a higher frequency program may be necessary to increase group cohesion in small groups and effectively improve exercise performance and social engagement.

Although the study contributes to the literature by demonstrating how higher program frequency, rather than program duration, may be vital to improving exercise performance and social engagement and maximizing the effects of high group cohesion in small groups, it also has several limitations. Being a nonrandomized controlled trial, it can introduce bias (30). Participant self-selection bias can be another limitation since participation was voluntary, and only those willing to exercise were included. Future work should include a randomized controlled trial to compare the effectiveness of this CEE program directly. In this study, the sample size was calculated after the study, but not a priori, using data from the primary outcomes CS-30, grip strength, and LSNS-6, as this information is necessary for planning the next study. This resulted in the need for 4, 6, and 927 participants in each group, respectively. Thus, future planned programs similar to this study would require smaller sample sizes for the primary outcome of exercise performance, such as CS-30 and grip strength, and larger sample sizes for the outcome of social participation.

In summary, a CEE program with a longer period and half the number of participants than our previous CEE program improved physical performance but did not affect social engagement, suggesting the need for a higher program frequency, rather than a longer duration, for improving exercise performance and social engagement. A higher frequency program can help maximize the effects of high group cohesion in small groups on exercise performance and social engagement. Further studies are needed to develop more effective interventions to extend healthy life expectancy.
